# Social Communication in Big Brown Bats

**DOI:** 10.3389/fevo.2022.903107

**Published:** 2022-06-16

**Authors:** Jessica Montoya, Yelim Lee, Angeles Salles

**Affiliations:** 1 Department of Biological Sciences, University of Illinois at Chicago, Chicago, IL, United States,; 2 Department of Psychological and Brain Sciences, Johns Hopkins University, Baltimore, MD, United States

**Keywords:** communication, bats (Chiroptera), social behavior, vocalizations, auditory processing

## Abstract

Bats are social mammals that display a wide array of social communication calls. Among them, it is common for most bats species to emit distress, agonistic, appeasement and infant isolation calls. Big brown bats (*Eptesicus fuscus*) are no different: They are gregarious animals living in colonies that can comprise hundreds of individuals. These bats live in North America and, typically found roosting in man-made structures like barns and attics, are considered common. They are insectivorous laryngeal echolocators, and while their calls and associated brain mechanisms in echolocation are well-documented, much less is known about their neural systems for analyzing social vocalizations. In this work we review what we know about the social lives of big brown bats and propose how to consolidate the nomenclature used to describe their social vocalizations. Furthermore, we discuss the next steps in the characterization of the social structure of this species and how these studies will advance both research in neuroethology and ecology of big brown bats.

## INTRODUCTION: THE LIFE HISTORY OF BIG BROWN BATS

Big brown bats, as most other bats, are gregarious and live in colonies that can range from dozens to hundreds of individuals. They are one of the most ubiquitous bats in North America, found from southern Canada to Central America, and in some Caribbean islands (Kurta et al., 1990).

**FIGURE 1 |:**
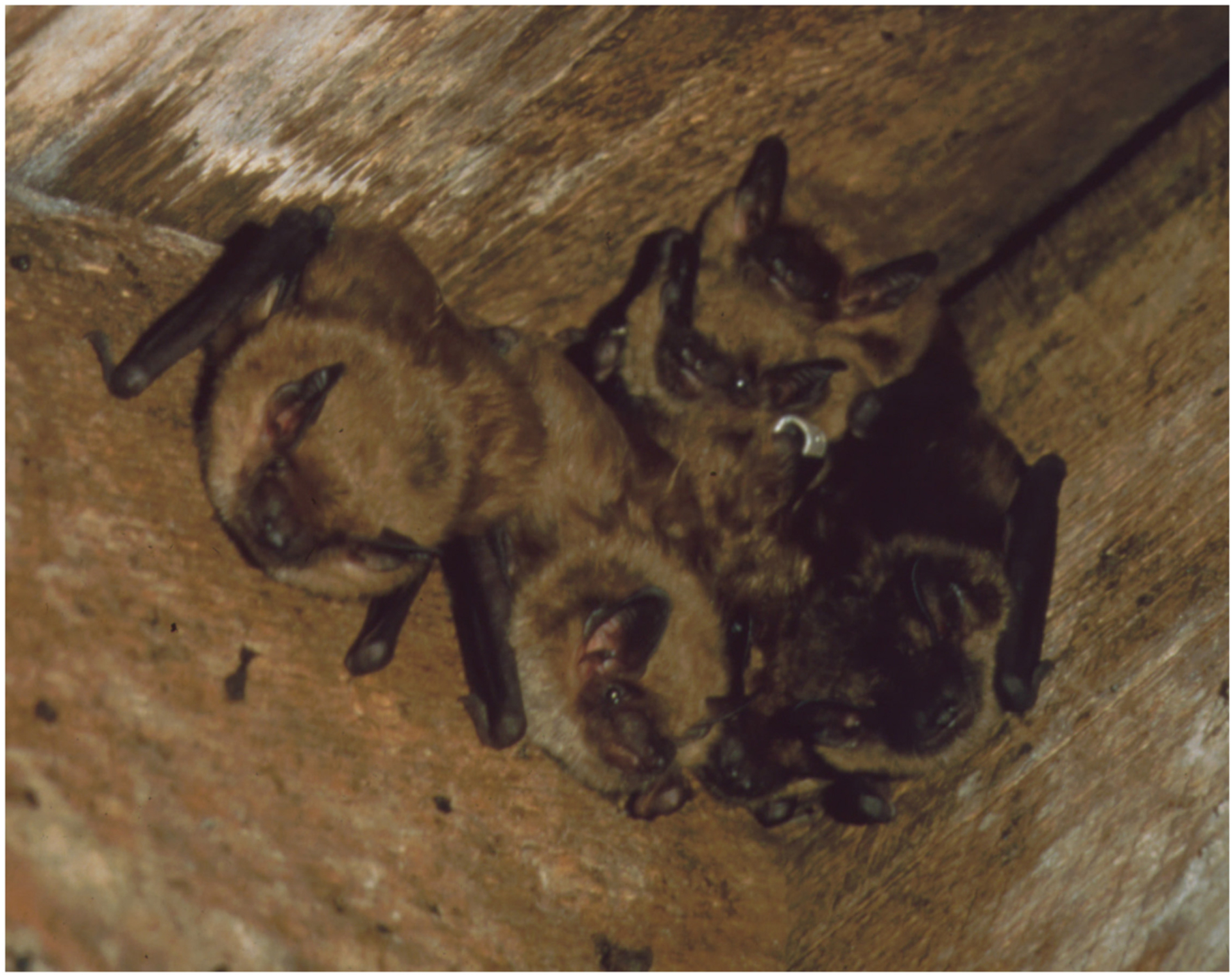
Huddle of at least 7 *Eptesicus fuscus* bats in a home attic. Note the banded individual at the center. Photo courtesy of Dr. Brock Fenton.

In summer, these bats prefer to roost in crevices of trees and man-made structures like barns and attics where they form their maternity colonies (Lausen and Barclay, 2006; Rancourt et al., 2007; Figure 1). Big brown bats are promiscuous, with males and females having multiple sexual partners. There is a delay between copulation and fertilization in big brown bats that is utilized both to await the favorable conditions of spring and also for post-copulatory sexual selection to occur (Vonhof et al., 2006). So, even though they mate in the fall, their pups are born in the spring. Female big brown bats usually give birth once a year, having up to two pups per cycle. Bat pups are born without fur and gain the ability of flight at about 4 weeks of age. Until then, the females leave their young while they go out foraging and locate them again upon their return. To locate and nurse their pups, females follow individually distinct isolation calls emitted by their own offspring (Rasmuson and Barclay, 1992). Female bats will likely return to the same colony in which they were born, while male bats leave the colony during their first fall (Willis et al., 2003). Big brown bats reach sexual maturity at 6 months of age for males and 1 year for females. In the fall when mating season begins, the colonies become mixed sex and this continues through hibernation (Agosta, 2002). Males of these urban and forest dwelling bats switch roosts every few days which is hypothesized to help with increasing the network of social connections and potential mates (Willis and Mark Brigham, 2004).

During winter, big brown bats go into hibernation and find roosting sites such caves, mines, or basements that are well-ventilated but remain above freezing and usually close to 10°C (Whitaker and Rissler, 1992). During hibernation, bats have been found either hanging alone or in mixed-sex clusters. The ability of these bats to form dynamic colonies that gather repeatedly across the years, even after switching roost across seasons, speaks to the complexity of their social interactions and the need for a highly sophisticated communication system.

## SOCIAL VOCALIZATIONS

Since echolocating bats rely on hearing as their main sensory modality, it is unsurprising that vocal communication is an important aspect of bat social communication. Adult big brown bats have a wide repertoire of communication signals which have been studied in detail (Gadziola et al., 2012a; Wright et al., 2013). The characterization and description of communication calls in big brown bats was pioneered by Gadziola and collaborators who described the vocalizations produced by bats while roosting and while engaged in different behavioral interactions (i.e., aggression and appeasement) (Gadziola et al., 2012a). Later, Wright and collaborators described social vocalizations emitted by free flying big brown bats engaged in a competitive foraging task (Wright et al., 2013). In conjunction, these studies suggest that the social vocalizations emitted by big brown bats are distinct across behavioral contexts and the repertoire only partially overlaps when comparing roosting and flying bats. As such, the vocal variety of big brown bats is rich in complexity and presents an opportunity to further study the underlying mechanisms for social vocalizations.

**FIGURE 2 |:**
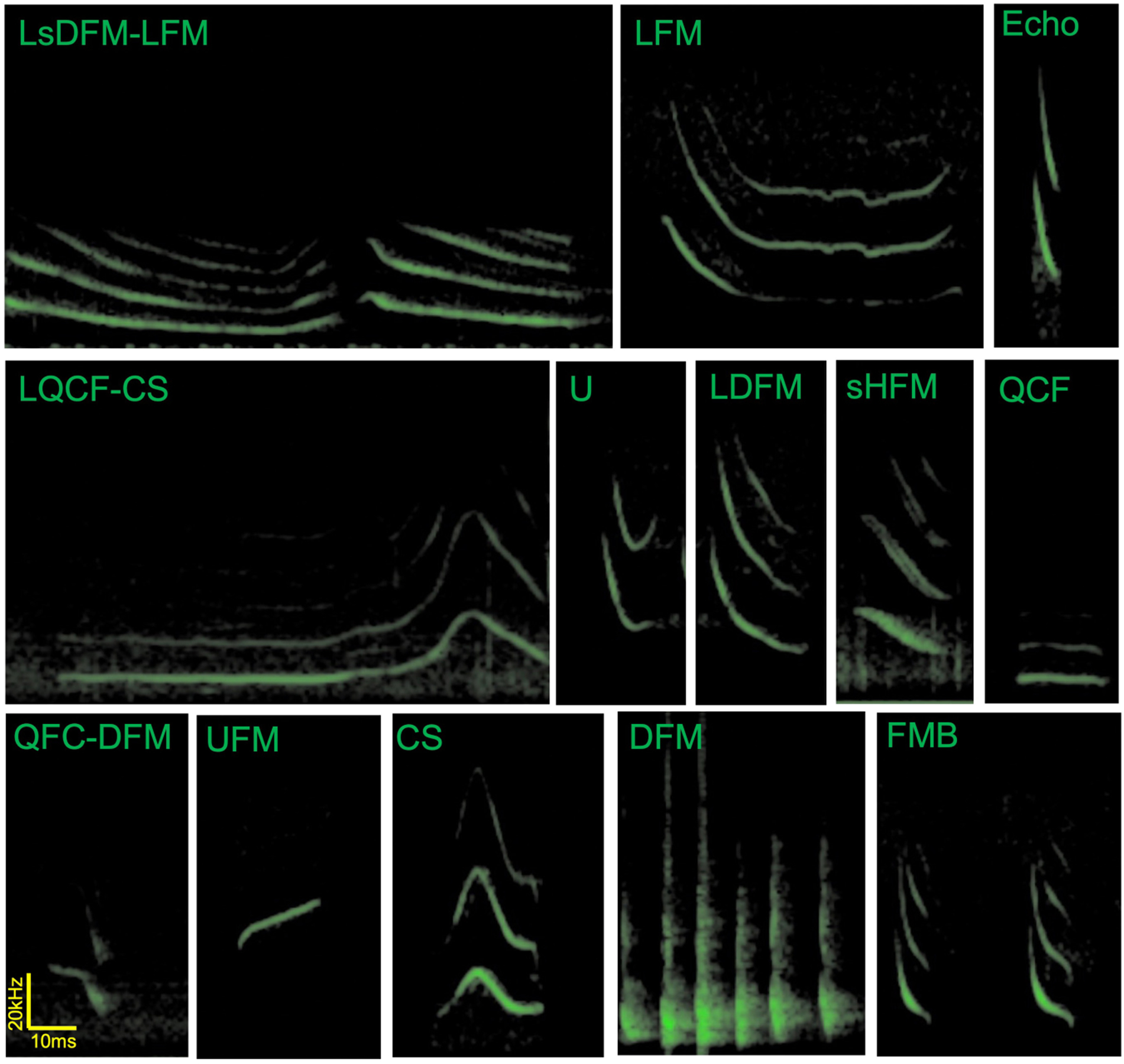
Example spectrograms of vocalizations by *Eptesicus fuscus* bats in flight and in paired interactions. These calls were recorded by Angeles Salles in Cynthia Moss’s laboratory at Johns Hopkins University. Note that social calls tend to have longer durations than echolocation calls (top right). LsDFM-LFM, long shallow frequency modulation downward—long frequency modulation; LFM, long frequency modulation; Echo, echolocation call; LQCF-CS, long quasi-constant frequency to chevron shape; U, U-shaped call; LDFM, long downward frequency modulation long; sHFM, single humped frequency modulation; QCF, quasi-constant frequency; QFC-DFM, quasi-constant frequency to downward frequency modulation; UFM, upward frequency modulation; CS, chevron shaped call; DFM, downward frequency modulation (here showing a bout containing 6 DFM syllables, these are often emitted as long bouts and may be audible to humans); FMB, frequency modulated bout (FMB refers to each syllable). Though presented separately in this figure, in our experience, U and LDFM calls are often emitted in close succession and at times as a continuum without clear separation of the syllables, similar to LsDFM-FM.

Unfortunately, these seminal papers describing the communication call repertoire of big brown bats do not share a consistent nomenclature to identify specific social vocalizations. Gadziola and collaborators describe calls of roosting bats in terms of the directionality of the frequency modulation which allows for building descriptive names for the calls—for example, QFC-DFM refers to a quasi-constant frequency section that continues into a downward frequency modulation (Gadziola et al., 2012a). On the other hand Wright and collaborators use descriptive words that refer to the perceived spectrogram of the calls of flying bats—for example, CS refers to a Chevron shaped call (Wright et al., 2013). Notably, some of the described calls may occur in both contextual situations, but these occurrences are rare. Both nomenclatures use the letter “L” to describe long versions of calls, though they do so in different ways (start/end and uppercase/lowercase). Here we propose the following points to reconcile the nomenclatures: (1) following the nomenclature established by Gadziola and collaborators in 2012 for the growing described number of syllables, including using the lower case letter “s” to describe shallow calls (to distinguish steep or shallow frequency modulation sweeps), (2) adding the duration descriptor when necessary at the beginning of the name in uppercase (S for short and L for long, following the S < 50 ms and L > 50 ms as suggested in Gadziola et al., 2012a), (3) adopting the established shape describing nomenclature for 6 of the 7 already described calls by Wright and collaborators in 2013 (changing SFM to DFM) and (4) using the nomenclature for single syllables and not multi-syllabic structures (i.e., FMB will now refer to each syllable in the multisyllabic bout). Figure 2 shows example spectrograms from vocalizations of big brown bats from the Johns Hopkins University (Maryland, United States) to illustrate the use of the consolidated nomenclature—for example, LQCF-CS refers to a long quasi-constant frequency followed by a chevron shape (note that not all calls described in Gadziola et al., 2012a; Wright et al., 2013 are present in this figure, nor are all calls in all stages of the bat’s life cycle). Standardizing the nomenclature across the literature will assist researchers as we explore further the social vocalizations of big brown bats.

Behavioral context is a major driver of the vocalizations produced by big brown bats. As in most other animals distress and agonistic calls are common, and display the stress or aggression of the animal in question when in these behavioral contexts, respectively. These calls are characterized by a “squawking” noise that is audible to the human ear and occurs frequently in interactions with conspecifics (Gadziola et al., 2012a). Big brown bats also produce appeasement calls that promote social contact between individuals. These calls are typically observed in bats that are jostling with each other in the roost or in bats being approached by other individuals (Gadziola et al., 2012a). Agonistic encounters can occur in flight too, while bats are foraging for food. One type of agonistic call that has been well- characterized in the big brown bat is the frequency modulated bout (FMB). These are individually distinct food claiming calls often emitted in sets of 3 or 4 syllables and currently only recorded from male big brown bats in-flight (Wright et al., 2013)—though anecdotal evidence suggests females may produce these calls too. A bat emitting an FMB will deter another bat that is in pursuit of the same prey item (Wright et al., 2014). It is possible that females are rarely observed emitting FMBs because they form non-kin relationships in the colony and are less likely to claim food when among roost mates (Wright et al., 2014). These calls are emitted as bouts, but it is still unknown to which extent individual syllable repetition in FMB is used to convey meaning. Other big brown bat calls also suggest that syllable combinations may be used to transmit information (for example, the repetition of DFM syllables, and U and LDFM calls often being emitted in close succession). Gadziola and collaborators explored and quantified the occurrence of multisyllabic structures in different behavioral contexts of roosting bats, and described simple multisyllabic calls as those containing repetitions of the same syllable and complex multisyllabic calls as those containing combinations of syllables (Gadziola et al., 2012a). In other bat species such as *Tadarida brasiliensis* several syllables are put together to form mating songs (Bohn et al., 2013). While specific mating calls have not been described yet in big brown bats, it has been found that male big brown bats’ echolocation calls change during the mating season to be distinct from female bats, serving as identification from possible mating partners (Grilliot et al., 2014). Although echolocation calls are not typically considered communication calls, they may be serving a function in information transfer. Further research is needed to explore the semantic structure that may arise through syllable combination in social vocalizations and its relation to behavior in big brown bats, and to investigate the extent to which echolocation calls may transmit information to aid social encounters such as mating.

Young big brown bats produce infant isolation calls that are used by mothers to find their pups in the colony after the mother has been out foraging for the night. Infant isolation calls are the only vocalizations produced by big brown bat pups until they are 4–6 days old (Gould, 1975a,b). After that, the pups begin to produce multiple vocalization types, many which start to resemble adult social communication calls (Moss, 1988; Monroy et al., 2011). This time during pup vocalization development in which they produce syllables that mirror those of the adult is defined as babbling (Monroy et al., 2011). In the sac-winged bat, compelling evidence supports the similarities of bat babbling with infant speech development (Fernandez et al., 2021), but this has not been studied in depth in big brown bats. Bats are one of the few groups of animals that have shown evidence of learned vocalizations (the others being humans, birds, pinnipeds, elephants, and cetaceans), yet, for big brown bats, it is still unknown to which extent they may learn their vocal repertoire from the adults. This new information and continued study of vocal learning in bats can shed light on the evolution of speech in humans, and provide another mammalian model to study social vocalization development.

## NEURAL PROCESSING OF COMMUNICATION CALLS

This wide repertoire of acoustic signals that big brown bats use for communication contains rich information ranging from physical properties to contextual features. Like other laryngeal echolocators, big brown bats have a highly developed auditory system that is sensitive to multiple characteristics of the sounds they perceive. This makes them ideal animal models to study the neural processing of communication sounds. Yet, most research on the neural mechanisms underlying auditory processing in these animals has focused on the processing of echolocation signals with fewer studies looking into the processing of social communication signals. Here we briefly review the work that has focused on big brown bats and the main regions of interest in the processing of social communication calls, these are the inferior colliculus (IC), the auditory cortex (AC), and to some extent the Amygdala (Amy).

The IC is the auditory hub in the midbrain that mediates the information ascending from the brainstem and relays it to the AC, while in turn also processing descending inputs from the AC. The IC has been an area of major focus for the study of echolocation as neuronal specializations aid the processing of subtle acoustic features of echoes. Fewer studies in big brown bats have focused on how the IC processes communication calls, yet the same neuronal specializations that govern the processing of echolocation can be co-opted for communication sounds. For example, the neuronal population of the IC of big brown bats includes frequency-tuned neurons that compute the spectral quality of the sounds and delay-tuned neurons that measure the latency between pulse and echo (Pinheiro et al., 1991; Casseday et al., 1997; Ehrlich et al., 1997; Fremouw et al., 2005; Thomas et al., 2012). Neurons in the IC of these bats are tonotopically mapped (Covey and Casseday, 1999) and show distinctive spike responses depending on the spectrotemporal properties of calls (Morrison et al., 2018; Salles et al., 2020). All these characteristics enable neuronal populations in the IC to be selective for specific communication call types, even when others may appear to be acoustically similar. For example, the IC of big brown bats contains neurons that are selective for FMB food claiming calls (described above), even though these resemble echolocation calls: FMBs and echolocation calls are both frequency-modulated downward sweeps sharing almost identical bandwidths, but, they differ in sweep rate, which we hypothesize to be the selectivity driver for these FMB selective neurons (Salles et al., 2020). This and work in other bat species supports the idea that the IC is also a center for the processing of communication calls. We aim to explore this further in the big brown bat, studying the IC as a hub for processing of social sounds and exploring how other brain areas interact to modulate responses at different levels depending on context.

Another area of interest is the auditory cortex, yet to our knowledge, there are no studies that explore the neurophysiological responses of AC neurons to communication sounds in the big brown bat. Comprising a large portion of the cerebral cortices of big brown bats, the AC functions as a precise analyzer of the auditory cues. Thus, we seek to explore communication call selectivity in the AC and the circuit mechanisms that may modulate selectivity in other areas such as the IC and amygdala. Studies of echolocation calls and echoes, and pure tone playbacks have revealed that sub-populations of AC neurons are specialized in frequency, echo delay, amplitude, and direction of the sound sources (Dear et al., 1993; Jen et al., 1997; Shen et al., 1997). Frequency maps of the bats are similar across individuals, while delay-tuned neurons (DTNs) are less tonotopically distributed in the AC (Dear and Suga, 1995). On the other hand, amplitude-shift neurons, a type of DTN, track the locations of sound sources by detecting changes in intensity while the sounds travel through the air (Dear and Suga, 1995). In big brown bats, sound stimuli are processed in the contralateral AC, following an ante-posterior tonotopic organization (Jen et al., 1997). Although not studied in detail, there are also ipsilateral connections that enable same hemisphere processing in the AC (Ma and Suga, 2001). However, studies also demonstrated that the AC neurons have certain levels of plasticity to alter their selectivity on different acoustic parameters depending on experience (Chowdhury and Suga, 2000; Gao and Suga, 2000), resulting in individual differences in the cortical maps. The information collected by isolated units is eventually integrated by combination selectivity that processes multifaceted aspects of acoustic stimuli (Kanwal and Rauschecker, 2007). These studies regarding the properties of neurons in the AC of big brown bats in response to different acoustic features, pave the way for our research avenue that will focus on communication calls and social behavior. We can leverage the neuronal population specializations to predict how the AC will respond to communication sounds, exploring patterns, and discrepancies with predictions to make hypotheses about circuit modulation driven by context.

The amygdala is a part of the limbic system that receives input from the auditory thalamus and the AC, among others. It guides context-related behaviors such as reward and motivation, fear conditioning, and defense mechanisms (Cardinal et al., 2002; LeDoux, 2007). Thus, this area has received some attention when exploring the neuronal responses to social communication in bats. For big brown bats, the amygdala is responsible of detecting vocal complexity and environmental context: Background discharge rates of the neurons in the basolateral amygdala (BLA) affects the responsiveness of neurons to social vocalizations, those with low background firing were found to be more selective than those with high background firing (Gadziola et al., 2012b, 2016). The sampling of BLA neurons may include a mixture of interneurons as well as principal neurons and different sampling procedures may affect the interpretation of amygdala responses to social signals (Wenstrup et al., 2020). Single neurons in the BLA also showed diverging spike rate and response duration depending on the emotional valence of behavioral situations such as aggression and appeasement (Gadziola et al., 2012b). This allows parallel auditory neural processing of communication and echolocation calls. We plan to build on these past studies and continue the research of the role of the amygdala in the processing of communication sounds in big brown bats and further explore how they can modulate information processing across the auditory pathway. With this battery of adaptations for auditory processing of natural sounds including communication and echolocation, big brown bats stand out as outstanding research animals to explore the pathways and mechanisms for auditory processing in the mammalian brain.

## OTHER FORMS OF COMMUNICATION

Despite the well-known adage, “blind as a bat,” bats can see even in poor light conditions (Ellins and Masterson, 1974). Insectivorous bats, such as the big brown bat, use sight to find their way out of a roost and to orient themselves (Bradbury and Nottebohm, 1969) and studies indicate that they can integrate vision with echolocation to aid navigation (Horowitz et al., 2004; Jones and Moss, 2021). While there is no current evidence that big brown bats use vision to communicate socially, this has been documented in other bat species: *Carollia perspicillata* will extend its tongue and shake its wings when displaying aggression, and *Epomophorus wahlbergi* performs a wing-flapping courtship display in which the male erects white tufts of hair (Fleming, 1988; Adams and Snode, 2015). *Centurio* senex bats perform wing-flapping displays and cover their faces with a skin flap during courtship behavior (Rodríguez-Herrera et al., 2020). Other bats have markings on their fur or bright colored noses that could play a role in the assessment of fitness during social interactions (reviewed in Chaverri et al., 2018). Further research is necessary in order to determine the possibility of visual social communication between big brown bats.

Olfaction is an important sensory modality in many species of bats. In fruit bats, olfaction helps bats identify food and in some, such as the sac winged bat, olfaction plays a large part in social interactions (Chaverri et al., 2018). Although anecdotal evidence suggests big brown bats produce strong smells when in aggressive or stressful situations, there is little evidence that they use scent to communicate. Female big brown bats of the same colony prefer the scent of roost mates compared to the scent of females from a different colony, with the scents of roost mates chemically resembling each other (Bloss et al., 2002). Yet, there is no evidence of big brown bat pups choosing the scent of their mother over the scent of other females (Mayberry and Faure, 2014). There is also no evidence that big brown bats distinguish between sexes based on olfactory cues, so it is believed that big brown bats use olfactory cues mainly to differentiate between colonies, not individuals (Greville et al., 2021). The colony scent differences could be due to the environment, such as the microbiota or microclimate of the hibernacula, or due to common food resources (Greville et al., 2021).

Lastly, as big brown bats most commonly roost huddled together, the possibility of tactile communication cannot be overlooked (Figure 1). In the close quarters of the roosts, bats groom each other—known as allogrooming—for hygiene and potentially for social functions. Both wild and captive big brown bats allogroom when roosting, yet there are few systematic studies focusing on tactile communication. Maternity colonies follow a fission-fusion model of roosting, where colonies form subgroups that differ from night to night, with big brown bats choosing roost mates non-randomly and not aligned with kin based patterns of association (Willis et al., 2005). Reciprocal allogrooming could be a social behavior that affects roosting decisions, as reciprocal allogrooming is observed in other species of bats. Furthermore, these bats utilize social thermoregulation, relying on the group’s body heat to keep individuals at appropriate temperatures while roosting (Willis and Brigham, 2007).

Big brown bats choose a familiar conspecific over an unfamiliar individual when tested in an alternative two choice task (Kilgour et al., 2013), yet, it is still unknown what exact combination of social cues and sensory modalities these bats are using to recognize each other.

## DISCUSSION

Because of all the characteristics reviewed here, big brown bats present an outstanding opportunity to study mammalian social interactions. They are abundant in North America and adapt well to laboratory life, which enables comparisons between field studies and those in a controlled environment. Lab experiments enable precise audio and video recordings of flying and roosting bats that may be restricted in natural colonies due to the inherent difficulty of recording in the field. In turn, field experiments help validate laboratory observations. For example, given territorial behaviors and roost-exiting patterns exhibited by these bats in the wild (Gillam et al., 2011) as well as anecdotal laboratory evidence, it is expected that big brown bats form hierarchical colonies, and we are only starting to study this systematically. This type of reciprocal studies will add to the knowledge of big brown bat ecology that may inform future conservation efforts for this species.

Easily kept in the lab and trainable, big brown bats also emerge as a remarkable model to study mammalian auditory processing of social sounds. Furthermore, the echolocating system of this efficient hawking insectivore presents the opportunity to comparatively study the auditory processing mechanisms involved both in echolocation and communication. Also, comparative studies across bat species will reveal specializations and commonalities across systems. Bats can bridge the gap between the wealth of knowledge acquired from the song processing system in birds and the psychophysical studies of language in humans. Though some studies reviewed here have already started to delve into understanding the social vocalization processing in these bats, there is still much to learn. Questions remain regarding the neural circuits and mechanisms that mediate the behavioral responses to social vocalizations and the role of behavioral context on the neural representation of these calls. Especifically, current and future studies of our group aim to follow up on the work by Marsh and collaborators (Marsh et al., 2002) in mustached and pallid bats to explore the existence of direct projections from the amygdala to the inferior colliculus of big brown bats and their role in the modulation of the neural responses to communication sounds in the inferior colliculus.

With this work we aim to provide background for these future studies and consolidate the nomenclature for the social vocalizations of big brown bats. We believe this will enable a better flow of information between research groups that aim to use these animals as mammalian models for social communication, and for those studying the ecology and evolution of big brown bats.
